# Aversive emotional interference impacts behavior and prefronto-striatal activity during increasing attentional control

**DOI:** 10.3389/fnbeh.2015.00097

**Published:** 2015-04-21

**Authors:** Apostolos Papazacharias, Paolo Taurisano, Leonardo Fazio, Barbara Gelao, Annabella Di Giorgio, Luciana Lo Bianco, Tiziana Quarto, Marina Mancini, Annamaria Porcelli, Raffaella Romano, Grazia Caforio, Orlando Todarello, Teresa Popolizio, Giuseppe Blasi, Alessandro Bertolino

**Affiliations:** ^1^Department of Basic Medical Sciences, Neuroscience and Sense Organs, University of Bari “Aldo Moro”Bari, Italy; ^2^IRCCS “Casa Sollievo della Sofferenza”, S. Giovanni RotondoItaly; ^3^Cognitive Brain Research Unit, Institute of Behavioral Sciences, University of HelsinkiHelsinki, Finland; ^4^Azienda Ospedaliero-Universitaria Consorziale, Policlinico di BariBari, Italy; ^5^pRED, NORD DTA Neuroscience, Hoffman-La Roche LtdBasel, Switzerland

**Keywords:** attentional control, emotion, inferior frontal gyrus, caudate nucleus, fMRI

## Abstract

Earlier studies have demonstrated that emotional stimulation modulates attentional processing during goal-directed behavior and related activity of a brain network including the inferior frontal gyrus (IFG) and the caudate nucleus. However, it is not clear how emotional interference modulates behavior and brain physiology during variation in attentional control, a relevant question for everyday life situations in which both emotional stimuli and cognitive load vary. The aim of this study was to investigate the impact of negative emotions on behavior and activity in IFG and caudate nucleus during increasing levels of attentional control. Twenty two healthy subjects underwent event-related functional magnetic resonance imaging while performing a task in which neutral or fearful facial expressions were displayed before stimuli eliciting increasing levels of attentional control processing. Results indicated slower reaction time (RT) and greater right IFG activity when fearful compared with neutral facial expressions preceded the low level of attentional control. On the other hand, fearful facial expressions preceding the intermediate level of attentional control elicited faster behavioral responses and greater activity in the right and left sides of the caudate. Finally, correlation analysis indicated a relationship between behavioral correlates of attentional control after emotional interference and right IFG activity. All together, these results suggest that the impact of negative emotions on attentional processing is differentially elicited at the behavioral and physiological levels as a function of cognitive load.

## Introduction

In everyday living, we perceive a complex visual environment and fully analyze many items and events at one time. Attentional control allows the flexible allocation of attentional resources to relevant stimuli while suppressing stimuli that are less relevant. This cognitive process provides a top-down bias for analysis and representation of relevant information in the face of concurrent and non-relevant information (Desimone and Duncan, 1995).

According to the load theory of attention, modulation of attentional processes by distractors during goal-directed behavior depends on the cognitive load (Lavie et al., 2004; Lavie, 2005). More specifically, processing of task-irrelevant information is dependent upon the perceptual attentional load in such a way that task-irrelevant information is processed only under low attentional conditions and is suppressed by high attentional loads. In other words, an increase in cognitive demands by active attentional processing may lead to greater inhibition of elaboration of distracting stimuli and to a reduced impact of these stimuli on behavior. This top-down regulation during goal-directed behavior may therefore be seen as a mechanism of hierarchical integration to ensure maintenance of performance at higher cognitive loads in the presence of potentially interfering stimuli (Gray et al., 2002; Serrien et al., 2006). However, the attentional load concept does not fully explain the divergent results reported in the literature, and it may be more flexible than initially thought. For example, facilitation effects on behavioral performance by task-irrelevant stimuli have been reported (Xu et al., 2011; Ziaei et al., 2014).

Emotional cues are critical during social interactions and can modulate cognitive processes, including attention. According to the “salience hypothesis,” modulation of attentional processes may also depend on emotional salience of task-irrelevant stimuli (Eltiti et al., 2005; Gupta and Srinivasan, 2015). For example, previous studies have indicated that negative, relative to neutral, emotional stimuli are associated with impairment of accuracy and reaction time (RT) during goal-directed attentional control processes (Simpson et al., 2000; Hare et al., 2005; Blair et al., 2007; Hindi Attar and Müller, 2012; Jasinska et al., 2012). On the other hand, some studies have reported facilitation of attentional processes during emotional interference (Geng and Diquattro, 2010; Swallow and Jiang, 2010; Kanske and Kotz, 2011; Lindström and Bohlin, 2011; Ziaei et al., 2014). Emotional and attentional paradigms in these studies varied both in terms of salience and attentional load, possibly explaining some of these inconsistencies. At the physiological level, the interaction between emotion and cognition is based on dynamic coordination between various brain areas that often participate in both emotion elaboration and cognition (Pessoa, 2008; Kellermann et al., 2012; Dolcos et al., 2013). For example, cognitive processes involving differentiation between targets and distractors and selection of appropriate responses have been associated with activity in prefronto-striatal circuits (McNab and Klingberg, 2008; Xu et al., 2011; Jarcho et al., 2014; Langeslag et al., 2014). Interestingly, in some recent studies, both inferior frontal gyrus (IFG) and caudate nucleus have been involved in the physiology of emotional interference during goal-directed attentional control processes (Langeslag et al., 2014; Ziaei et al., 2014). In particular, the IFG has been specifically associated with sustained attentional control, coding of behavioral significance of emotional stimuli, inhibition of distracting negative emotions, and regulation of motor responses (Aron et al., 2004; Ochsner et al., 2004; Phan et al., 2005; Beer et al., 2006; Dolcos and McCarthy, 2006; Erk et al., 2007; Sakagami and Pan, 2007; Mitchell et al., 2008; Sommer et al., 2008; Schulz et al., 2009; Urry et al., 2009; Hampshire et al., 2010; Mincic, 2010; Munakata et al., 2011; Shafer and Dolcos, 2012; Depue et al., 2015). In more detail, the orbital and triangular parts of the IFG are distinctly involved in resolution of emotional interference (Schulz et al., 2009; Levens and Phelps, 2010). The orbital part is the main area of the IFG to interface between sensory events and cognitive control (Sakagami and Pan, 2007; Kret et al., 2011). The opercular and triangular parts of the IFG are both involved in executive function and emotional interference (Schulz et al., 2009; Barber et al., 2013). However, these two areas are cytoarchitectonically different and functionally dissociable (Heim et al., 2009; Katzev et al., 2013). Furthermore, the caudate nucleus has been related to attentional processing, elaboration of negative stimuli, and related arousal (Crofts et al., 2001; Herwig et al., 2007; Roiser et al., 2007; Scholes et al., 2007; Carretié et al., 2009; Levita et al., 2009; Gerdes et al., 2010; Geliebter et al., 2013; Hart et al., 2013; Jarcho et al., 2014), as well as to integration of emotional and cognitive processing and suppression of emotional interference (Langeslag et al., 2014; Ziaei et al., 2014). Interestingly, IFG activation has been associated with emotional interference during processing of stimuli requiring low levels of attention, and caudate activation has been associated with emotional interference when high levels of attentional processing are required (Blair et al., 2007; Ali et al., 2010; Xu et al., 2011; Ziaei et al., 2014). However, there are no studies exploring how emotional interference modulates activity in these areas and related behavior during increasing levels of attentional control processing.

The aim of this study was to investigate the potential for emotional task-irrelevant items to modulate behavior and brain activity during various levels of attentional control. With this purpose, we used stimuli from a recently developed cognitive paradigm, the Variable Attentional Control (VAC) task, which requires increasing levels of attentional control processing associated with a physiological linear increase in prefrontal activity (Blasi et al., 2005, 2007, 2010, 2013; Zhang et al., 2007). The three levels of attentional control generated by the stimuli of this task were manipulated to study the relationship between varying cognitive load and emotional processing. Fearful and neutral facial expressions were used to add emotional interference for each level of attentional processing. Based on previous studies, we predicted that cognitive performance as well as activity in the IFG and in the caudate nucleus would vary as a function of the interaction between attentional load and emotional interference. According to recent findings, we also predicted that the IFG would be involved in the physiology of emotional interference at low levels of attentional control and the caudate nucleus at higher levels (Blair et al., 2007; Ali et al., 2010; Xu et al., 2011; Ziaei et al., 2014). Consistent with the load theory of attention, and given the greater potential for emotional interference of aversive, relative to neutral, stimuli, we also hypothesized that brain activity in these areas would be preferentially sensitive to negative emotional interference during lower loads of attentional control.

## Methods

### Subjects

Twenty-two healthy subjects were enrolled in this study (13 males; mean age ± SD, 25.5 ± 4.5 years). Inclusion criteria were absence of any psychiatric disorder, as evaluated using the Structural Clinical Interview for Diagnostic and Statistical Manual of Mental Disorders IV, of any significant neurological or medical condition revealed by clinical and magnetic resonance imaging evaluation, of history of head trauma with loss of consciousness, and of pharmacological treatment that could influence cerebral metabolism or blood flow, or drug abuse in the past year. The Wechsler Intelligence Scale—Revised was used to evaluate the intelligent quotient (IQ) (mean ± SD, 103.2 ± 14.5). Socio-economic status (Hollingshead and Redlich, 1955) (mean ± SD, 36.3 ± 16.2) and handedness (Oldfield, 1971) were also measured (mean ± SD, 0.74 ± 0.4). All subjects underwent fMRI while performing the Emotional VAC task (see below).

Written informed consent was obtained from all participants prior to enrolling them in the study, which received approval from the Independent Ethical Committee of “Azienda Ospedaliero-Universitaria Consorziale, Policlinico di Bari”. All experimental procedures were carried out in accordance with the 2013 WMA Declaration of Helsinki.

### Emotional Variable Attentional Control (EVAC) Task

**Figure 1:**
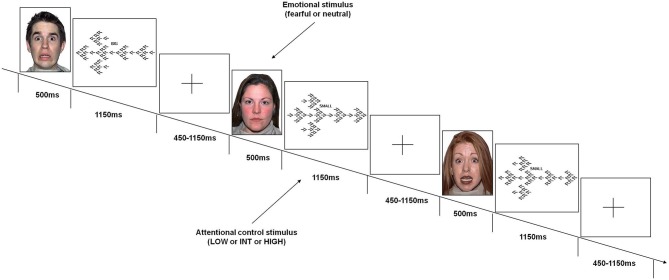
**Emotional variable attentional control (EVAC) task**. Events included emotional stimuli (fearful or neutral faces) before stimuli requiring three levels of attentional control load (LOW or INT or HIGH).

Subjects performed a task (Figure 1) that was specifically designed to obtain emotional interference preceding various demands of attentional control processing. Attentional stimuli used in this task were identical to those used in previous studies (Blasi et al., 2005, 2007, 2010, 2011, 2013; Zhang et al., 2007). These stimuli consisted of arrows of three different sizes (1 large, 6 medium, 42 small) pointing either to the right or to the left; seven small arrows were embedded in each medium-sized arrow; and six medium-sized arrows were embedded in one large arrow. The direction of the arrows of each size was always the same. To increase the level of attentional control required, the direction of the stimuli of each size was congruent or incongruent relative to those of other sizes. This resulted in the following conditions:

- Low level of attentional control (LOW): All three sizes of arrows were congruent in direction with each other. The cue was the word BIG.
- Intermediate level of attentional control (INT): The big arrow was incongruent in direction to the small and the medium arrows; the cue was the word SMALL.
- High level of attentional control (HIGH): The medium sized-arrows were incongruent in direction to the big and the small arrows; the cue was the word SMALL.

Emotional interference was obtained using unfamiliar faces with either fearful or neutral facial expressions derived from a validated set of facial pictures (NimStim; Tottenham et al., 2009).^1^ These emotional stimuli were displayed just before the attentional processing stimuli without any time interval.

Subjects were instructed by a cue word (BIG or SMALL) displayed above each attentional stimulus to press a button corresponding to the direction of the large or small arrows (either right or left). They were instructed to respond to the attentional stimuli with the thumb of their right hand using a button box (right button for “right” response, left button for “left” response), and to press the response button as fast and accurately as possible. Furthermore, they were asked to move their thumb to a small plastic knob placed between the buttons after each response. All subjects were trained on attentional stimuli of the task prior to the fMRI session to stabilize performance. More specifically, just before fMRI scanning, subjects were instructed on task rules and performed the task outside the fMRI environment until the average behavioral performance did not grossly vary across trials in terms of behavioral accuracy (±10 percentage points across trials). No instructions related to emotional stimuli were administered.

A total of 192 attentional stimuli was preceded by the respective 192 stimuli providing emotional interference, which were 96 fearful and 96 neutral facial expressions. There were 64 stimuli for each level of attentional control. Thirty two stimuli of each attentional level were preceded by facial stimuli with fearful expression, and 32 by facial stimuli with neutral expression. This design resulted in 6 conditions: fearful-low attentional control; fearful-intermediate attentional control; fearful-high attentional control; neutral-low attentional control; neutral-intermediate attentional control; neutral-high attentional control. The order of the stimuli was randomly distributed across the session (Friston et al., 1999). Each emotional stimulus was presented for 500 ms, while each attentional stimulus was displayed for 1150 ms. The total duration of the task was 8 min. A fixation cross-hair was presented during the interstimulus interval (before emotional stimuli), which ranged from 450–1150 ms (mean ISI = 700 ms).

Stimuli in the fMRI setting were presented via a back-projection system and responses were recorded through a fiber optic response box, which allowed measurement of RT for each trial. We report behavioral performance of the task performed in the scanner during the fMRI experiment.

### Blood Oxygen Level-Dependent fMRI

We performed BOLD fMRI using a GE Signa 3T scanner (gradient-echo-planar-imaging sequence, time repetition/time echo = 2000/30 ms; 26 interleaved slices, thickness = 4 mm, gap = 1 mm; voxel size = 3.75 × 3.75 × 5 mm; scans = 260; flip angle = 90°; field of view = 24 cm; and matrix = 64 × 64) while subjects performed the emotional variable attentional control (EVAC) task. The first 4 scans were discarded to allow for signal saturation.

### Data Analysis

#### Behavioral Data

Analysis of variance (ANOVA) was used to compare behavioral data (% correct responses and RT of correct responses). We included only subjects who had an average accuracy above 50% (forced choice between two options). Bonferroni correction was used for* post hoc* analysis to correct for multiple comparisons. Because of our priori hypothesis, *post hoc* analysis of the interaction between the level of attentional control and emotional interference was performed for each level of attentional control between fearful and neutral faces.

#### fMRI Data

Analysis was completed using the event-related module within Statistical Parametric Mapping 5 (SPM5).^2^ Images of each subject were realigned, spatially normalized into the Montreal Neurologic Institute (MNI) template (a 12-parameter affine model), and spatially smoothed (10-mm Gaussian filter). After realignment, data sets were also screened for high quality (scan stability) as demonstrated by small motion correction (less than 2.5 mm translation and less than 2° rotation). The fMRI responses were modeled using a canonical hemodynamic function and temporally filtered using a high-pass filter of 128 Hz and an HRF-shape low-pass filter. Vectors were created for each condition using the timing of correct responses for each stimulus type. The timing of incorrect responses and residual movement were also modeled as regressors of no interest. A *t* statistic was then used to produce a statistical image for BOLD responses relative to brain processing of stimuli associated with correct responses for each condition. A random effects ANOVA was used at the group level to investigate the main effect of emotional interference, of attentional control stimuli, and their interaction. For all analyses on activity during the EVAC task, we focused our attention on the orbital and triangular parts of IFG and on the caudate nucleus because of their central role in the regulation of cognitive and emotional processes as well as in their integration. Thus, we used a statistical threshold of *p* < 0.05, *k* ≥ 5, Family Wise Error (FWE)-corrected for multiple comparisons within brain regions of interest (i.e., orbital and triangular parts of IFG, left and right caudate), as defined by the WFU pickatlas software, version 1.04 (Functional MRI Laboratory at the Wake Forest University School of Medicine).^3^ The orbital and the triangular parts of the IFG were used as two distinct regions of interest (ROIs) because of their different cytoarchitectonic features, connections with different brain areas, and different functional contributions (Petrides and Pandya, 2002; Wimber et al., 2008). Although both areas are involved in coding of behavioral significance of emotional stimuli and both exert control over attentional and emotional function regulation needed to perform the task (Sakagami and Pan, 2007; Schulz et al., 2009), only the triangular part appears to be involved in complex sensory guided motor acts, possibly by storing motor representation of goal-directed hand actions (Iacoboni and Wilson, 2006).

To further investigate directionality of the interaction between emotional interference and attentional load, *post hoc* analysis was performed on parameter estimates extracted from clusters crossing the statistical threshold of *p* < 0.05 (FWE corrected) within the predefined ROIs using MarsBar.^4^ Finally, to investigate the relationship between brain activity and behavior, Spearman’s correlation analysis was performed between parameter estimates extracted with the same threshold from the above-mentioned ROIs and both accuracy and RT during the EVAC task.

## Results

### Behavioral Data

ANOVA on accuracy data indicated a main effect of increasing level of attentional control (*F*_(2,42)_ = 31.25; *p* < 0.000001; average number and % of correct responses: HIGH 26.55, 82.9%; INT 29.52, 92.3%; LOW 31.32, 97.9%), no effect of emotional interference (*F*_(1,21)_ = 0.98; *p* = 0.3), and no interaction between level of attentional control and emotional interference (*F*_(2,42)_ = 1.37;* p* = 0.2). *Post hoc* analysis of the main effect of increasing level of attentional control showed a significant difference across all three levels (HIGH > INT, *p* < 0.00004; HIGH > LOW, *p* < 0.0000001; INT > LOW, *p* < 0.02).

**Figure 2:**
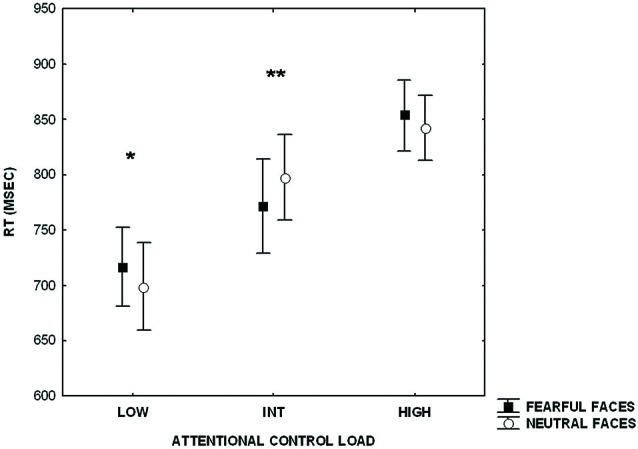
**Plot showing reaction time (RT)s during processing of attentional control stimuli (LOW, INT, HIGH) after emotional stimuli (fearful and neutral faces)**. Measures presented are mean ± standard deviations. *p < 0.002, **p < 0.002.

Furthermore, analysis of RTs revealed a main effect of increasing level of attentional control (*F*_(2,42)_ = 103.25; *p* < 0.000001), no effect of emotional interference (*F*_(1,21)_ = 0.07; *p* = 0.7), and an interaction between level of attentional control and emotional interference (*F*_(2,42)_ = 14.23; *p* < 0.00002) (Figure 2). *Post hoc* analysis of the main effect of increasing level of attentional control showed a significant difference across all three levels (HIGH > INT, *p* < 0.0000001; HIGH > LOW, *p* < 0.0000001; INT > LOW, *p* < 0.0000001). *Post hoc* analysis of the interaction between the level of attentional control and emotional interference indicated a statistically significant difference between RTs at the lower level of attentional control when preceded by presentation of fearful facial expressions (fearful > neutral, *p* < 0.002). An effect of emotional interference was also present at the intermediate level of attentional control, but in the opposite direction (neutral > fearful; *p* < 0.002). At the higher level of attentional control, a statistical trend in the same direction of the lower level was found (fearful > neutral; *p* = 0.06).

### Imaging Data

**Figure 3:**
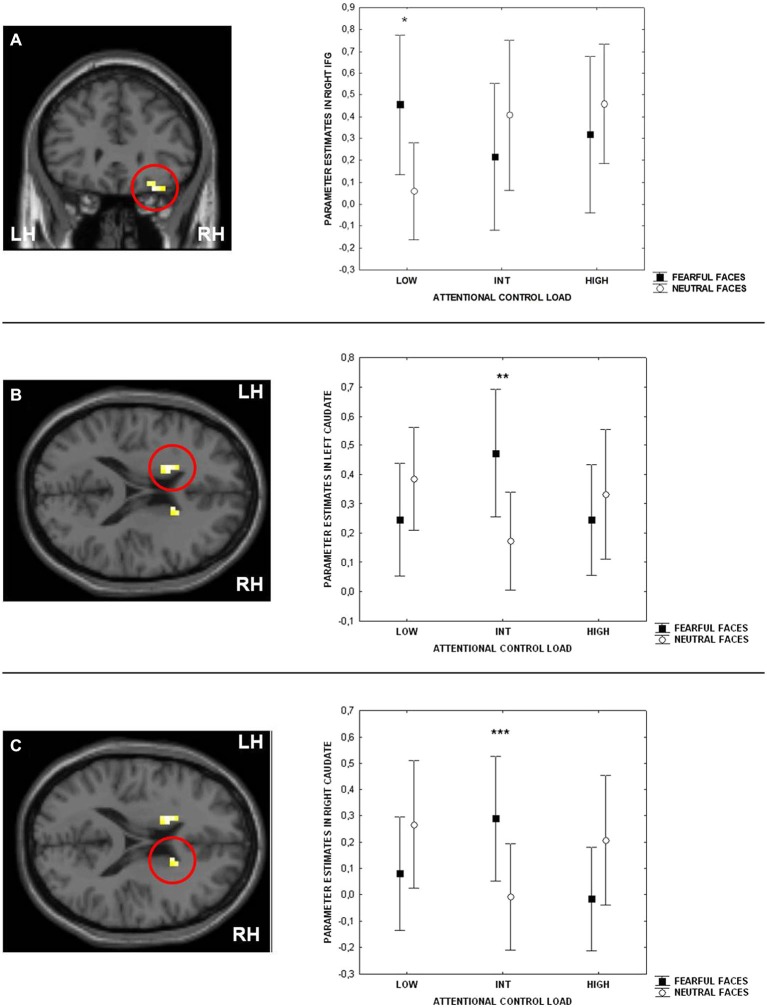
**Sections of the brain showing the interaction between attentional control load and emotion in right Inferior Frontal Gyrus (IFG) (BA47) (A), left (B) and right (C) caudate**. Graphs represent parameter estimates extracted from these clusters. Measures presented are mean ± standard deviations. *p = 0.004, **p = 0.001, ***p = 0.01. For more statistics, see Section Results.

Consistent with previous studies (Blasi et al., 2005, 2007, 2010), ANOVA indicated a main effect of attentional control load in the right side of the IFG (BA9 and BA47) (Table 1). No effect of emotional interference was found on brain activity. However, there was a significant interaction between the level of attentional control and emotional interference in the right side of the IFG (BA47) and in both sides of the caudate (Figure 3; Table 1). *Post hoc* analysis of parameter estimates extracted from IFG revealed that interference by fearful facial expressions at the low level of attentional control was associated with greater activity relative to interference with neutral facial expressions (*p* = 0.004) (Figure 3A). Similar *post hoc* analyses for the left and right sides of the caudate again indicated an effect of emotional interference in the same direction, but at the intermediate level of attentional control (left:* p* = 0.001; right:* p* = 0.01) (Figures 3B,C). A statistical trend in the opposite direction was found for the right side of the caudate at the higher level of attentional control (*p* = 0.06).

**Table 1:** **Local maxima of brain activity associated with a main effect of attentional control and with an interaction of attentional control by emotion**.

		Talairach coordinates		
Brain region	BA	*x*	*y*	*z*	*k*	*Z*
		**Activity**
**Main effect of attentional control load**
Right Inferior Frontal Gyrus	9	49	16	20	34	4.36
Right Inferior Frontal Gyrus	47	34	26	−7	30	3.99
**Interaction: attentional control load by emotion**
Right Inferior Frontal Gyrus	47	30	28	−20	5	3.34
Left Caudate		−19	9	17	16	3.29
Right Caudate		15	12	19	5	3.17

### Correlation Analysis

**Figure 4:**
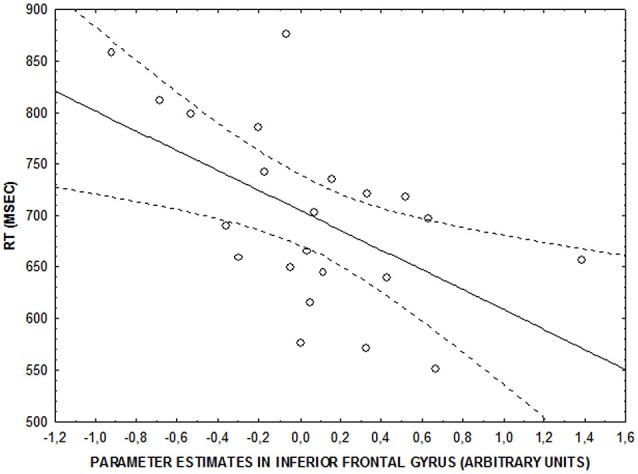
**Scatterplot showing the correlation between activity in right IFG and RT (ms) at the lower level of attentional control after neutral faces (*r* = −0.5; *p* = 0.009)**.

No significant correlations were found between behavioral accuracy data and brain activity in clusters showing an emotional interference by attentional load interaction (all *p* > 0.2). However, there was a significant negative correlation between parameter estimates in right IFG and RTs at the low level of attentional control after neutral facial expressions were shown (*r* = −0.5; *p* = 0.009) (Figure 4). No significant correlation was found at the same level of attentional control after fearful facial expressions were shown (*r* = −0.33; *p* > 0.1). Finally, no significant brain activity-RT correlations were found at the intermediate and high levels of attentional control (*p* > 0.15).

## Discussion

Our results indicated differential effects of emotional interference on behavior and activity in prefronto-striatal brain areas during attentional control as a function of cognitive load. In particular, stimuli eliciting the lower level of attentional control were associated with slower RTs as well as greater activity in the right side of the IFG when anticipated by fearful, relative to neutral, facial expressions. However, the interaction between emotional stimuli and the intermediate level of attentional control was associated with the opposite behavioral pattern but with a similar effect on brain activity, this time taking place in both sides of the caudate. Finally, no statistically significant behavioral or imaging effects of emotional interference were found at the higher level of attentional control. Together, these findings suggest that negative emotions modulate goal-directed behavior and related brain activity when low and intermediate levels of attentional control are required, but they do not impact attentional processing when high levels of attentional control are required. More generally, these results suggest that negative emotions may modulate cognitive efficiency during attentional control processing.

At the lower level of attentional control, the presentation of fearful, relative to neutral, faces before attentional stimuli was associated with longer RTs. Slower performances of attentional processes by negative emotions has been previously reported (Vuilleumier et al., 2001; Mather et al., 2006; Blair et al., 2007; Jasinska et al., 2012). Moreover, our data support that this slowing is related to the emotional salience of interference and the level of attentional control required for goal-directed behavior. In more detail, slowing of cognitive processes takes place when negative emotional stimuli anticipate goal-directed behavior requiring a low level of attentional control. This result coincides with the load theory of attention and the “salience hypothesis.” According to the first model, when lower attentional control levels are directed to task-relevant items, task-irrelevant stimuli result in high interference because sufficient cognitive resources are available to process all information. Furthermore, the “salience hypothesis” suggests that this interference depends on the emotional salience of task-irrelevant stimuli. We found that task-irrelevant negative emotions have a detrimental effect on RTs when low levels of attentional control were directed to task-relevant items. Moreover, our fMRI data are also consistent with earlier reports, indicating greater activation in the right side of the IFG after fearful facial expressions were presented at the lower level of attentional control only (Blair et al., 2007; Xu et al., 2011). In particular, we found greater engagement of activity on the right side of the IFG for slower responses, suggesting a greater need for neuronal resources when negative emotional interference is present. Moreover, correlation analysis revealed that greater responses in this brain area after neutral facial expressions were presented during low levels of attentional control were correlated with faster RTs, while this relationship is not as tight after fearful facial expressions were presented. A possible interpretation of these results is that the physiological relationship between activity in right IFG and RT during attentional processing partially breaks down after presentation of stimuli with high emotional salience, such as viewing fearful expressions. Therefore, our results may support greater engagement of the IFG to control the impact of aversive facial expressions only when a low attentional level is required and that at the intermediate level, this interference is no longer regulated by cortical areas, but by subcortical ones.

At the intermediate level of attentional control, anticipation of attentional stimuli by fearful, relative to neutral, faces was associated with faster RTs. Facilitation of attentional processes by negative emotional items has been reported previously (Erk et al., 2007; Geng and Diquattro, 2010; Swallow and Jiang, 2010; Kanske and Kotz, 2011; Lindström and Bohlin, 2011; Ziaei et al., 2014). Moreover, our data suggest that this facilitation is related to the emotional salience of distracters and the level of attentional control required for goal-directed behavior. In particular, cognitive processes were facilitated when negative emotional stimuli anticipated goal-directed behavior requiring intermediate levels of attentional control. A possible interpretation of this result is that when intermediate levels of attentional control are required during goal-directed behavior, sufficient cognitive resources are still available to process task-irrelevant information. In line with recent studies, arousal elicited by the processing of task-irrelevant negative emotions may increase attentional control efficiency during goal-directed behavior (Lindström and Bohlin, 2011; Sutherland and Mather, 2012). We found that task-irrelevant negative emotions had a beneficial effect on behavior when the intermediate level of attentional control was directed to task-relevant items. Also, our fMRI data indicated greater activity in the right and left sides of the caudate after fearful facial expressions were presented at intermediate level of attentional control. These data coincide with a recent study suggesting the key role of this brain area when irrelevant stimuli interfere at higher levels of attentional control (Ali et al., 2010). Moreover, caudate activity has been associated with automaticity during cognitive processes and higher behavioral efficiency (Raichle et al., 1994; Delazer et al., 2003; Floyer-Lea and Matthews, 2004; Poldrack et al., 2005; Saling and Phillips, 2007; Harsay et al., 2011; Ofen et al., 2012). However, we found that a signal change in right and left sides of the caudate failed to correlate with behavioral responses during the task. Still, in a recent study, activation in the caudate nucleus has been correlated with better behavioral performance after negative emotional interference at higher levels of attentional processing (Ziaei et al., 2014). Therefore, our data may suggest additional recruitment of neuronal resources in the right and left sides of the caudate to filter interference of aversive facial expressions during intermediate levels of attentional control. Another, more speculative, interpretation is that filtering out of emotional interference by caudate activation leads to faster RTs. These explanations are speculative and should be treated with caution.

Finally, at the higher level of attentional control there were no significant differences between fearful and neutral interference in behavioral and imaging data, even though statistical trends were present. This lack of any interference by task-irrelevant stimuli only at higher levels of attentional control has been previously reported (Sadeh and Bredemeier, 2011). Our data suggest that goal-directed behavior requiring high levels of attentional control is relatively independent of any emotional interference especially if compared with the effects found at the lower and intermediate levels. A possible interpretation of this result, based on the load theory of attention, is that at higher attentional levels, task-irrelevant information does not achieve significant interference because of possible saturation of all available resources during processing of task-relevant information.

Another possible speculation from our results may take into account putative arousing effects associated with aversive stimuli (Erk et al., 2007; Gotoh et al., 2010). Some previous studies have demonstrated that the greater the arousal elicited by emotional stimuli, the slower the RTs achieved during cognitive processing (Hartikainen et al., 2007; Kuhbandner and Zehetleitner, 2011; Ossowski et al., 2011). However, in other studies arousal has been associated with the opposite behavioral pattern or no impact on RTs (Lindström and Bohlin, 2011; van Steenbergen et al., 2011). Taken together, our results could suggest that greater arousal elicited by negative emotional stimuli rather than neutral ones is able to modulate goal-directed behavior. Furthermore, this modulation depends on the level of attentional control required for processing of task-relevant items with detrimental and beneficial effects at the lower and the intermediate levels respectively, and no interference at the higher levels.

Finally, our data may suggest that the signaling function (alertness) induced by fearful facial expressions modulates detection of other environmental cues. Similar results have been reported recently (Phelps et al., 2006; Bocanegra and Zeelenberg, 2009; Gable and Harmon-Jones, 2010; Lv et al., 2011). According to our results, fearful faces may reduce the speed of perception and inhibit detection of other environmental cues when low loads of attentional control are required. On the other hand, when intermediate levels of attentional control are required, fearful facial expressions could increase speed of perception and facilitate detection of other environmental cues. These observations fit well with the view that the adaptive function of emotional states is to rapidly and flexibly switch between different modes of response to best meet the current challenges of the environment (Gray, 2004; Sutherland and Mather, 2012).

A limitation of this study may be the number of trials used at the high level of attentional control, which could explain the statistical trend we found in behavioral and imaging data between fearful and neutral interference. According to a previous study, 25 trials are sufficient to provide stable activation maps (Murphy and Garavan, 2005). We found more than 25 correct trials for each condition, on average, at the high level of attentional control (26.6 or 83.1% with fearful interference and 26.5 or 82.8% with neutral interference). This is the reason why we did not expect any influence from the number of trials on our results.

In conclusion, this study suggests that emotionally irrelevant stimuli during attentional control processing modulate behavior and brain activity in right IFG and both sides of the caudate as a function of cognitive load. These findings add knowledge to some aspects of the complex functional architecture underlying the integration of emotion and attention in the human brain and could have potential clinical implications. Impairment of emotional and cognitive functions have long been regarded as characterizing several major psychiatric disorders such as schizophrenia, bipolar disorder, depression, and anxiety disorders (Bertolino et al., 2006, 2009; Matsuo et al., 2007; Siegle et al., 2007; Pauly et al., 2008; Sailer et al., 2008; Blasi et al., 2009, 2010; Etkin et al., 2010; Lee et al., 2010; Sadeh and Bredemeier, 2011; van Wingen et al., 2011; Brotman et al., 2014; Diwadkar et al., 2014). However, little is known about the interaction between emotion and cognition in patients. As such, our findings could provide a basis for formulating and experimenting hypotheses in future research aimed at investigating the nature of neural deficits in these disorders and their clinical correlates.

## Conflict of Interest Statement

Dr. Bertolino is a full time employee of Hoffman-La Roche, Ltd. The authors declare that the research was conducted in the absence of any commercial or financial relationships that could be construed as a potential conflict of interest.

## Footnotes

^1^http://www.macbrain.org/resources.htm

^2^http://www.fil.ion.ucl.ac.uk/spm

^3^http://rad.wfubmc.edu

^4^http://marsbar.sourceforge.net/

## Abbreviations

IFG: Inferior Frontal Gyrus
EVAC: Emotional Variable Attentional Control.
